# Understanding Repetitive Behaviours: A clinical and cost-effectiveness, multi-site randomised controlled trial of a group for parents and carers of young autistic children

**DOI:** 10.1177/13623613251333175

**Published:** 2025-06-09

**Authors:** Victoria Grahame, Ashleigh Kernohan, Ehsan Kharati, Ayesha Mathias, Chrissie Butcher, Linda Dixon, Sue Fletcher-Watson, Deborah Garland, Magdalena Glod, Jane Goodwin, Saoirse Heron, Emma Honey, Ann Le Couteur, Leila Mackie, Jessica Maxwell, Lewis Montgomery, Emmanuel Ogundimu, Helen Probert, Deborah Riby, Priyanka Rob, Leanne Rogan, Laura Tavernor, Luke Vale, Elspeth Imogen Webb, Christopher Weetman, Jacqui Rodgers

**Affiliations:** 1 Cumbria, Northumberland, Tyne and Wear NHS Foundation Trust (CNTW) UK; 2 Newcastle University, UK; 3 Durham University, UK; 4 The University of Edinburgh, UK; 5 The National Autistic Society, Newcastle Upon Tyne, UK; 6 NHS Lothian, UK; 7 Tees, Esk and Wear Valleys NHS Foundation Trust (TEWV), UK

**Keywords:** autism spectrum disorders, interventions – psychosocial/behavioural, pre-school children, school-age children

## Abstract

Restricted and repetitive behaviours vary greatly between autistic people. Some are a source of pleasure or create opportunities for learning; others may be detrimental in day-to-day life or cause harm. We have developed, in close collaboration with parents/carers, the Understanding Repetitive Behaviours programme, designed for families of young autistic children, to help them recognise, understand and respond sensitively to their child’s impactful restricted and repetitive behaviours. This study is a clinical and cost-effectiveness, multi-site randomised controlled trial of the Understanding Repetitive Behaviours parent programme versus a psychoeducation programme (equivalent to current best practice), learning about autism. Participants were parents/carers, with an autistic child aged between 3–9 years and 11 months. The study was delivered across three sites in England and Scotland. Analyses were completed using intention-to-treat principles. Two hundred and twenty seven families were randomised (113 in LAA; 114 in Understanding Repetitive Behaviours arm). No differences were found between the arms on the primary outcome measure (The Clinical Global Impression – Improvement scale). Analysis of secondary outcomes indicated that children in the Understanding Repetitive Behaviours arm were more likely to be rated as responders in target impactful restricted and repetitive behaviours at 24 weeks but that this effect was not maintained at 52 weeks. Improvements in parent and family functioning were apparent, with no evidence of differences between the arms. The study reconfirms that it is important that clinicians consider both restricted and repetitive behaviours and social communication needs of autistic children with parents when planning appropriate support.

**Lay abstract:**

Autistic children, frequently repeat the same behaviours over and over, have specific interests or like things to stay the same. These behaviours and interests are often fun and helpful. However, sometimes they can impact negatively on day-to-day life or put the child at risk of harm. Working closely with parents of autistic children, we developed an 8-week programme (Understanding Repetitive Behaviours) to help them recognise and understand these behaviours. This study aimed to find out whether the understanding repetitive behaviour programme was helpful and good value for money. Two hundred and twenty seven families were allocated by chance to receive either Understanding Repetitive Behaviours or a learning about autism programme. When experts made judgements about whether children showed positive changes across various measures, and these were analysed, there were no differences between the programmes. However, parents who attended the Understanding Repetitive Behaviours programme reported improvement in one of their child’s specific repetitive behaviour (selected to be the main focus of the Understanding Repetitive Behaviours programme) at 24 weeks after the end of the programme. Parents who attended either programme reported more confidence, greater wellbeing and less stress up to 1 year after the end of the study.

## Introduction

Restricted, repetitive, and stereotyped patterns of interests, behaviours and activities (RRB) are an aspect of everyday life for autistic people and are frequently reported to be enjoyable, helpful, functional, and pleasurable (Cho et al., 2017; Collis et al., 2022; Grove et al., 2018; Manor-Binyamini & Schreiber-Divon, 2019). There are many reported positive impacts of RRB such as a coping mechanism to help self-regulate and manage sensory and emotional arousal and as an expression of passionate interests (e.g. collections, hobbies). Many autistic children and young people report that they wish teachers would use their special interests to facilitate their learning (i.e. as a tool to support their engagement) (Mullally & Connolly, 2023). For some autistic people, interests may lead to employment (Leekam et al., 2011), increase opportunities for social interaction (Koegel et al., 2013) and provide a basis for friendship and community (Kim & Bottema-Beutel, 2019). RRB can support skill development and be a valuable mechanism to improve an autistic individual’s ability to function and cope with their environment (Bakan, 2014b; Manor-Binyamini & Schreiber-Divon, 2019). RRB should therefore be acknowledged as an important aspect of neurodivergence (Bakan, 2014a) and the pathologising of RRBs resisted (Kapp et al., 2019).

However, some RRB may cause difficulties for young autistic children. Their presence may put the child at risk of physical harm or make it difficult to complete everyday activities, perhaps interfering with learning or reducing participation in day-to-day activities (Cunningham & Schreibman, 2008; Hayes & Watson, 2013; Leekam et al., 2011). RRB may also be an outward sign of anxiety or distress, deserving of attention and care from others (Leekam et al., 2011). Sometimes RRB can develop into harmful and extreme patterns of behaviour. For example, a widening gap between the autistic child’s behaviour and that of their non-autistic peers or siblings might mean caregivers try to limit the child’s access to their preferred RRB. All these different situations require people around the autistic child – especially caregivers – to understand and respond to children’s RRB in ways that promote acceptance and facilitate autonomy and development. We know that families can find understanding this repertoire of behaviours particularly difficult, with parents spontaneously identifying RRB as the most challenging aspect of parenting their autistic child (Honey et al., 2008) and, as a result, parents are often unsure how best to support their child. Parents often report that during the early years they do not receive specific professional advice on how to recognise, understand and differentiate their child’s potentially harmful RRB (hereby known as impactful RRB) from those that are not harmful (Hodgson et al., 2018). For all these reasons, it is important to identify evidence-based, effective and efficient interventions to support parents of young autistic children to understand how to respond to impactful RRB.

In close collaboration with parents of autistic children, utilising an ‘active’ research model, the Understanding Repetitive Behaviours (URB) parent programme was designed and developed. The aim of the parent programme was to support parents of young autistic children to recognise, understand and respond sensitively to their child’s negatively impactful RRB (Grahame et al., 2015). URB achieves this by increasing parents understanding of their child’s RRB and supporting them to develop strategies to differentiate between RRB that are beneficial or pleasurable for their child and those that are harmful. Parents have in-depth expert knowledge of their own child’s development and behaviours across a range of settings and are well placed to deliver new forms of support (McConachie & Diggle, 2007).

This study reports the findings of a large-scale randomised controlled trial (RCT) of the clinical and cost-effectiveness of the URB programme delivered in existing community settings by multi-agency professionals with expertise in working with young autistic children and their families. Group leaders were trained to deliver either the URB or an attentional-control psychoeducation programme (equivalent to current best practice) – learning about autism (LAA).

## Methods

### Study design

This study is a clinical and cost-effectiveness, multi-site, two-group, RCT of the URB parent group programme versus a psychoeducation parent group (equivalent to current best practice), LAA for parents of young children aged 3–9 years 11 months in the United Kingdom. Assessments were administered at baseline entry to the trial, at the end of the eight parent group sessions (10 weeks from start of group), at the 24-week primary endpoint and at the 52-week follow-up (from start of parent group) (for further information, please see published protocol paper (Grahame et al., 2021))

### Study aims and objectives

1. Compare the clinical effectiveness of the URB intervention for NHS community clinical practice with a psychoeducation parent group (equivalent to current best practice), for the management of negatively impactful RRB in autistic children at 24 and 52 weeks follow-up.
2. Assess the cost-effectiveness and cost consequences of the URB intervention compared with an autism psychoeducation parent group (LAA; LAA equivalent to current best practice) at 52 weeks follow-up.

### Sample size calculation

The initial sample size calculation was based on a type I error rate of 5%, and intra-cluster correlation of 10%. Assuming a 20% improvement rate and 90% power, it resulted in 14 parental groups per arm with 8 families per group (224 families). Allowing for an attrition rate of 12%, a minimum of 250 families was required to be randomised (125 randomised to each arm). The assumption of 10% intra-parental-group correlation was based on a review of group programmes in education trials (Lord et al., 2012). Sample size was calculated in R using n4props in Sample Size Estimation Functions for Cluster Randomised Trials (CRTSize) package (Sparrow et al., 2005).

### Statistical analysis plan

All analyses were done under intention-to-treat principle. The primary outcome at 24 weeks used Generalised Estimating Equation (GEE) with binomial distribution and logit link to compare the proportion of autistic children with improved global improvement between the URB group and the LAA group. The GEE method accounted for the clustering of children by parent groups using exchangeable working correlation. The continuous secondary outcomes were analysed using difference-in-difference models based on linear mixed-effects models accounting for repeated measurements per child and clustering of children by parent groups. All binary or categorical secondary outcomes were analysed using GEEs. We also performed safety and sensitivity analysis for missing data and assessed the impact of the COVID-19 pandemic on primary and secondary outcomes. The results of the sensitivity analyses for missing data (where a weighted GEE was used) and the impact of COVID showed that there was no significant difference between the two arms for Clinical Global Impression (CGI). The impacts of the COVID-19 pandemic that started during the course of this trial were assessed on the primary outcome and Target Behaviour Vignette (TBV) using difference-in-difference models between the participants who had their 24-week (primary endpoint) visit pre- and post-COVID-19 lockdown starting on 23 March 2020. This was specifically done by creating an indicator variable which takes values of 1 (‘Post-lockdown’) for data collected after 23 March 2020 and values of 0 (‘Pre-lockdown’) for the data collected before the lockdown. Although this study was not powered to detect interactions, a significant interaction between lockdown indicator and URB programme status would indicate that the outcomes differ between the pre- and during lockdown periods.

### Economic analysis

Three economic evaluations were undertaken: a cost-effectiveness analysis (CEA) where the costs are compared to the children who reach the target CGI-I, a cost utility analysis (CUA) where costs are compared to quality adjusted life years (QALYs) and a cost consequence analysis (CCA) where costs are compared to a balance sheet of all the measured outcomes (Drummond et al., 2015). The base case economic evaluations were carried out from a National Health Service (NHS) perspective over the time horizon of 1 year. An alternative analysis included time and travel costs associated with attendance to appointments for the child and their caregiver.

Costs were based upon use of services by the child over the 52-week follow-up. These costs related to the costs of the intervention itself and use of secondary care and community-based services as well as any medication costs (a detailed description of how this was performed is reported in the Supplementary Material). All costs are reported in 2020 Great British Pounds (GBP).

The primary outcome of the CEA was incremental (i.e. the extra cost) per additional person meeting the target difference in the CGI-I at 24 weeks. For the CUA, the primary outcome of the CUA was the incremental cost per QALY at 52 weeks with QALYs estimated for caregivers using responses to the European Quality of Life 5 Dimensions Level Version quality of life instrument and separately using QALYs estimated for the child based on proxy responses provided by the caregiver using the CHU9D instrument (Devlin & Krabbe, 2013; Ratcliffe et al., 2012) using the tariff by Stevens (2012). For the CCA, the costs are compared with the primary and secondary outcomes from the trial using a balance sheet approach (Mauskopf et al., 1998).

### Estimation of effects

#### Clinical Global Impression – Improvement scale outcomes

The primary effectiveness outcome measure for the cost-effectiveness analysis is achieving at least the target difference in Clinical Global Impression – Improvement scale (CGI-I) at 24 weeks. The outcome is expressed as a percentage of children who achieved their targeted outcome. As noted above, the results for the CEA are expressed as the incremental cost per additional child reaching their target improvement in the CGI-I outcome.

#### QALY outcomes

The primary measure of effects for the CUA is the QALY derived from responses to the CHU9D for the children and using the EQ-5D-5L for the caregivers as a measure of their own health-related quality of life. These instruments were both measured at baseline, 24 and 52 weeks. The EQ-5D-5L was scored using the EQ-5D-3L value set using the van Hout crosswalk (Van Hout et al., 2012). The CHU9D was completed by a proxy on behalf of the child as a measure of their health-related quality of life. More information about the CUA is included in the Supplementary Materials.

### Participants

Participants were parents/carers aged 18 and over, with an autistic child between 3–9 years and 11 months. All had sufficient spoken and written English, were willing to be randomised and attend all programme sessions and agreed to maintain their child’s current medication up to 24 weeks and not to participate in any other trials up to 24 weeks.

### Measures

#### Baseline characterisation and outcome measures

The clinical effectiveness of this intervention was assessed by measuring whether children showed global improvement using the CGI-I scale (primary outcome) at 24 weeks (primary endpoint) after parents attended the URB programme compared with the children whose parents had attended the LAA programme. The secondary outcome measures including both independent, teacher and parent-reported measures were collected at the end of group (10 weeks) and at two follow-up points (24 and 52 weeks) (see Table 1 for details). The measures were chosen in collaboration with parents/carers to assess: (1) changes in parents knowledge and skills in understanding their child’s impactful RRB; (2) changes in levels of child impactful RRB, and any changes in participation in everyday activities, levels of parent stress and impact on family life; (3) whether or not the new URB intervention provides value for money for the NHS and (4) whether any improvements are maintained at 1 year from baseline. There is additional information provided below on the CGI-I and the TBV in terms of the procedure applied in this study.

**Table 1.** Primary and secondary child, health economic and family outcome measures.

Participant type	Measure–PRIMARY	Time point (baseline, 10, 24, 52 weeks)
	Clinical Global Impression–Improvement scale (CGI-I; Guy, 1976). A standardised framework using a 7-point scale, ranging from very much improved to very much worse to assess a child’s baseline functioning. The CGI-I was rated by independently trained clinical researchers, blind to group allocation, who utilised all available child information from baseline, week 10 and 24 assessments (SRS-2, RBQ-2, Teacher RBQ-2, VABS-3 and target RRB vignettes). In line with other published studies, ratings of 1 (very much improved) and 2 (much improved) are regarded as clinically significant ‘improvement’ (Bearss et al., 2015).	Baseline and 24 weeks
Parents/carers	Incremental cost per QALY gained for the child: Child Health Utility 9D instrument (CHU9D; Stevens, 2012). A paediatric generic preference-based measure of health-related quality of life for this patient group. Scores used to create utility values as an average incremental cost per QALY.	Baseline, 24 and 52 weeks
Participant type	Measure – SECONDARY	Time point (baseline, 10, 24, 52 weeks)
Parent/carers	Social Responsiveness Scale – Second Edition (SRS-2; Constantino & Gruber, 2012). 65-item measure of social and communication features, characteristic of autism.	Baseline only
	Repetitive Behaviour Questionnaire – 2 (RBQ-2; Turner, 1996). 20-item questionnaire measuring the frequency and intensity of RRB.	All
Participant type	Measure – SECONDARY	Time point (baseline, 10, 24, 52 weeks)
Parents/carers	Vineland Adaptive Behaviour Scales 3 (VABS 3); (Sparrow et al., 2016). A measure of aspects of the child’s level of adaptive functioning: communication, daily living skills, socialisation, and motor skills.	Baseline and 24 weeks
	Target Behaviour Vignette (TBV) (Arnold et al., 2003). A measure of the duration, impact and possible triggers and functions of negatively impactful RRBs.	All
	Autism Family Experience Questionnaire (AFEQ) (Leadbitter et al., 2018). A measure of the broader impact on young autistic children and their families in participation in everyday activities.	Baseline, 24 and 52 weeks.
	Parent self-efficacy (PSE) (Sofronoff & Farbotko, 2002). 15-item self-report measure. A higher mean self-efficacy score indicates more behaviours displayed by the child over a period.	All
	Autism Parenting Stress Index (APSI) (Silva & Schalock, 2012). A measure of parenting stress specific to core and co-occurring features of autism.	All
	Warwick–Edinburgh Mental Wellbeing Scale (WEMWBS) (Tennant et al., 2007). 14-item questionnaire.	Baseline, 24 and 52 weeks.
Participant type	Measure – SECONDARY	Time point (baseline, 10, 24, 52 weeks)
Parents/carers	QALYs for the caregiver: The EQ-5D-5L (Van Reenen et al., 2014). A standardised instrument for use as a measure of health outcome. Responses used to create utility values to create QALYs for caregivers as part of a cost consequence analysis.	Baseline, 24 and 52 weeks
	Resource use questionnaire. A bespoke questionnaire used to measure the use of health care resources (e.g. GP or outpatient appointments), used to calculate the average cost of services for each intervention.	Baseline, 24 and 52 weeks
	Time and travel questionnaire. A bespoke questionnaire used to measure the time and travel costs to the family related to URB.	Baseline, 24 and 52 weeks
	Demographic information collected pertaining to the child and parents/carers.	Baseline only
Teachers/educational professionals	The Teacher Repetitive Behaviour Questionnaire–2 (Teacher RBQ-2) (Johnson et al., 2017). The corresponding version of the parent RBQ-2 for completion by teachers/educational professionals.	All

##### CGI-I (Guy, 1976)

CGI-I (Guy, 1976) was completed at 24 weeks follow-up. The CGI-I is a well-recognised standardised measure of clinically meaningful change. The CGI-I is a 7-point scale designed to measure overall improvement from baseline. Scores range from 1 (very much improved) through 4 (unchanged) to 7 (very much worse). The CGI-I was rated by a clinical researcher, blind to group allocation and trained to reliably rate global improvement and how much the child’s negatively impactful RRB had changed over the 24 weeks (from baseline to primary endpoint). The information contributing to the CGI-I ratings included comparison of all available child information from baseline, the end of group to week 24 (primary endpoint). The CGI-I has been used as the primary outcome measure in previous autism trials (Bearss et al., 2015). The majority of studies have used scores of 1 (very much improved) or 2 (much improved) to define positive response, although some studies have used 3 (minimally improved) with all other scores indicating negative response. In this study, we will use scores of 1 (very much improved) or 2 (much improved) to define a positive response. For 10% of participants, the global improvement score was independently rated by a second trained researcher to assess inter-rater reliability.

##### TBV

The TBV (Arnold et al., 2003) asked parents at baseline to identify two negatively impactful RRB. Parents were asked to describe the duration, impact and possible triggers and functions of these two negatively impactful RRB, using a standardised semi-structured interview. The protocol for measuring change in the parent-defined TBV was originally developed by The Research Units on Paediatric Psychopharmacology and Psychosocial Intervention Programmes (RUPP Autism Network). At each outcome assessment point, the parent/carer completed the follow-up version of the standardised semi-structured interview. The parent responses at each time point were audio recorded and contributed to a vignette written by the research associates (RAs). In keeping with the procedure developed by RUPP, after all data were collected, a panel of blinded autism experts independently rated change in each target behaviour and change in relation to the impact on the family. Three pairs of vignettes (comparing each time point (10; 24; 52 week) to baseline) were rated for each child on a 9-point scale of improvement/deterioration (1 – very much improved; 2 – markedly improved; 3 – definitely improved; 4 – equivocally improved; 5 – no change; 6 – equivocally worse; 7-definitely worse; 8 – markedly worse; 9 – disastrously worse). A positive response (also described as a ‘responder’) was defined as a rating of 3 or less. This procedure has demonstrated high levels of agreement between expert raters, with an intra-class correlation coefficient (ICC) of 0.895 across a panel of five raters (Arnold et al., 2003). For this study, each rater was trained in the vignette ratings procedure to achieve reliability and a shared understanding of the scoring definitions of each of the different ratings. Each vignette pair was then rated by four raters and the average rating was calculated. A limitation of this study is that inter-rater reliability was not undertaken on the final ratings.

### Recruitment, trial oversight, blinding and randomisation

Families were identified through clinicians in three sites (Edinburgh, Teesside and Newcastle). Clinicians were asked to introduce the study to families and study information packs were also sent out through diagnostic clinic databases and research databases such as autism spectrum disorder database–UK (ASD-UK). Expression of Interest (EoI) forms were returned to the research team who then contacted parents to provide more information about the study and arrange baseline assessment appointments. To minimise the risk of contamination, the RA was based on separate university premises to the group programme leaders and was to group allocation. Randomisation was at child level using an equal allocation ratio. The trial had two independent oversight committees; the Trial Steering Committee and Data Management Committee, which were independent of the sponsor and funder and declared no competing interests. The Statistical Analysis Plan (SAP) and Health Economic Analysis Plan (HEAP) were both shared prior to analysis with our Trial Steering Committee and Data Management Committee. We can confirm that there were no changes to pre-specified analysis plans. This trial received ethical approval on 20 August 2018 from the South West – Plymouth and Cornwall Ethics Committee: 18/SW/0173.

### Parent groups

#### URB programme

URB was developed with the aim to help caregivers of young autistic children to recognise, understand and learn how to respond to their child’s impactful RRB. It is an 8-week manualised parent-mediated programme designed to be delivered by two trained multi-agency professionals with knowledge and experience of working with young autistic children and their families. Each weekly session lasts for approximately 2 h (total duration ~16 h). Each parent/carer is provided with a manual, related weekly materials and given individual support to review their chosen impactful RRB and identify strategies to address impactful RRBs. This target negatively impactful RRB is then the focus for parents to practise the new skills they are learning, thus ensuring that strategies are individually tailored for each child. Parents are supported during the group to explore developmental trajectories of autistic children, review their understanding of RRBs in Autism, investigate types of RRBs, develop a functional framework approach for the ‘target’ negatively impactful RRB, explore the potential communicative function of RRBs, consider behavioural and environment responses that might be relevant for the ‘target’ negatively impactful RRBs, use video and group support to assess impact for child and promote generalisation of skills. The types of situations selected by parents included examples of risk of injury, risk of choking and risk of nutritional deficiency due to restricted diet.

The URB programme includes three key stages:

1. Help parents understand why their child might engage in a particular behaviour and identify behaviours that may indicate distress and/or are most likely to have a functional impact.
2. Support parents to identify and apply developmentally appropriate and sensitive strategies for responding to selected, functionally impactful RRB, including adjusting or modifying potential situational stressors or facilitating communication between parent and child.
3. The key aim is to help parents understand and promote their child’s opportunities to develop an extended repertoire of positive coping strategies to deal with the stressors that may have previously triggered a particular RRB.

The URB programme was designed to work with and alongside parents in a group setting. Parents have in-depth expert knowledge of their own child’s development and behaviours across a range of settings. By providing parents with information and understanding about RRB, the aim is to help them to support their child to extend their own behavioural repertoire in different contexts. Furthermore, the intervention aims to both provide parents with the skills to understand their child’s impactful RRB and through sharing this experience with the other parents in the group, promote parental wellbeing and family resilience through an increase in their sense of competence, a reduction of stress and an improvement in family cohesion. This in turn should have benefits for the child (for further detail on the development of the URB programme, assessment of feasibility and piloting of the URB programme prior to this more recent study, please see (Grahame et al., 2015) and (Hodgson et al., 2018)).

#### LAA programme

LAA is an 8-week manualised caregiver psychoeducation programme that acted as an attentional control. It is designed for caregivers of young autistic children and focuses on understanding autism and what that means for their child. It is designed to be delivered in the community by one trained professional with experience of working with autistic children and their families. Each weekly session lasts for approximately 2 h (total duration ~16 h). These sessions were delivered by a group leader trained and approved by the National Autistic Society, a UK-based autism charity.

The key difference between the two parent programmes is that the LAA programme focuses more generally on providing information about autism and does not include any specific information about the role and functions of RRB, or tailored strategies to respond to impactful RRB.

### Recording and reporting serious adverse events

This study recorded both serious adverse events (SAEs) and Events of Special Interest (ESI) for the parent and child participants. SAEs and ESI were recorded from the start date of participation in either LAA or URB until the follow-up assessment at week 24.

### Impact of COVID-19

Recruitment to and participation in the study were impacted by the onset of the COVID-19 pandemic in March 2020. Recruitment was terminated at the Edinburgh site but continued at Teesside and Newcastle, in accordance with requirements from differing oversight bodies in Scotland and England. For enrolled participants, the study remained open at all sites, necessitating a number of procedural changes: all consent and assessment visits moved to remote delivery (via telephone) and both programmes were delivered online using secure digital platforms. The Autism Diagnostic Observation Schedule-Second Edition (ADOS-2) assessment was removed as a baseline measure for new participants.

### This trial received ethical approval on 20 August 2018 from the South West – Plymouth and Cornwall Ethics Committee: 18/SW/0173

#### Community involvement statement

In close collaboration with parents of autistic children, utilising an ‘active’ research model, the URB parent programme was designed and developed. Parents have further contributed to many aspects of the research including feedback on assessment measures, how the research was conducted and dissemination of our findings. This collaborative working has greatly informed many aspects of the design of URB, from training materials to the acceptability of the programme, piloting the feasibility of outcome measures and the use of video feedback as a strategy for working on an agreed target impactful RRB. The proposed study parent advisory group was not possible due to the pandemic restrictions, but three parents were recruited to the Trial Steering Committee (unfortunately one parent withdrew due to other commitments). Two co-applicants on the study are parents of autistic children and one of these is also a parent support advisor with the National Autistic Society. After the trial was completed, we established a dissemination working group with two parents of autistic children and four autistic individuals with expertise in research and clinical practice (three of whom are also parents of autistic children) to help us consider how to proactively and positively engage with the autism community in relation to the interpretation and dissemination of the study findings. The group met on four occasions over a 6–month period, online and discussed: (1) getting to know each other, and URB including an open Q&A session on the programme and its rationale; (2) language for talking about RRB, the URB programme and reporting on the trial; (3) audiences for informal and formal outputs – who will be interested, how we can reach them and answer their questions or concerns? And (4) clinical implications of the research and lessons learned from the study.

## Results

### Clinical effectiveness analysis

**Figure 1.**
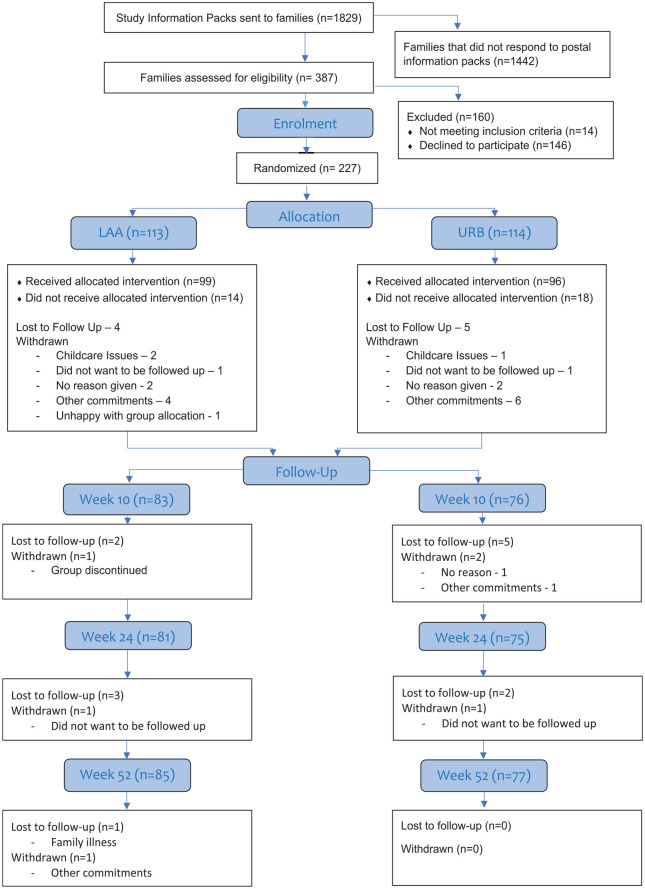
The CONSORT diagram and the recruitment and retention to the trial.

Figure 1 shows the participant flow through the study (consort diagram). The first family was randomised on 13 November 2018 and the final family was randomised on 15 September 2020. The study consented 262 participants and 227 participants were randomised (113 in LAA and 114 in URB). A minority of families (n = 47) completed their participation in the trial, up to and including the 24-week (primary outcome) follow-up assessment, prior to the onset of COVID-19 restrictions. This meant that only 25% of families recruited to the study experienced the trial in its original research design format. As a consequence of missing data, largely exacerbated by the pandemic, 155 families have usable data at 24 weeks for the primary endpoint.

Table 2 provides a summary of all the baseline demographics for the child.

**Table 2.** Baseline child demographic characteristics.

Variable	LAA			URB		
	N (Missing)	Mean (SD)	Range	N (Missing)	Mean (SD)	Range
Age	113 (0)	6.23 (1.74)	3.25-9.83	114 (0)	6.16 (1.66)	3.03-9.96
Female	22/113 (19.47%)			24/114 (21.05%)		
Male	91/113 (80.53%)			90/114 (78.95%)		
Missing	0/113 (0%)			0/114 (0%)		
Child ethnicity n/N (%)						
Any white background	94/110 (85.45%)			94/111 (84.68%)		
Black/ African/ Caribbean/Black British	7/110 (6.32%)			7/111 (6.32%)		
Asian	5/110 (4.55%)			6/111 (5.42%)		
Any other ethnic group	4/110 (1.82%)			4/111 (1.8%)		
Missing/prefer not to say	3/113 (2.65%)			3/114 (2.63%)		

N = number of available data.

A summary of all the baseline measures at child level is provided in Table 3.

**Table 3.** Child baseline measures.

Variable	LAA			URB		
	N (Missing)	Mean (SD)	Range	N (Missing)	Mean (SD)	Range
SRS-2	101 (12)	83.9 (7.96)	57-90	102 (12)	82.83 (7.71)	57-90
RBQ-2 mean total	109 (4)	2.15 (0.33)	1.3-2.9	109 (5)	2.09 (0.35)	1.35-2.9
RBQ-2 motor sensory	110 (3)	2.16 (0.43)	1.25-3	109 (5)	2.06 (0.44)	1.25-3
RBQ-2 insistence on sameness	109 (4)	2.1 (0.44)	1-3	110 (4)	2.09 (0.43)	1.33-3
RBQ teacher mean total	63 (50)	1.8 (0.41)	1-2.75	68 (46)	1.69 (0.33)	1.05-2.55
VABS 3 communication	106 (7)	66.82 (18.51)	32-135	105 (9)	66.78 (15.38)	32-121
VABS 3 socialisation	100 (13)	66.87 (9.17)	47-90	104 (10)	68.49 (8.95)	47-91
VABS 3 daily living skills	106 (7)	70.53 (14.03)	32-115	103 (11)	71.36 (11.34)	48-106
VABS 3 ABC score	97 (16)	67.4 (9.99)	42-111	98 (16)	67.85 (8.47)	51-96

The values of the mean total scores for SRS-2 and VABS-3 were similar in both arms. The range of scores reported for the SRS-2 (57-90) and the VABS-3 ABC (42-111) suggests a sample of autistic children with mixed abilities, including children with and without a co-occurring intellectual disability.

Table 4 provides key baseline characteristics of the main parent/caregiver who attended the parent group programme. Further details can be found in the funder report (Grahame et al., 2024).

**Table 4.** Baseline characteristics of parent/carer.

Variable	LAA n/N (%)	URB n/N (%)	Total n/N (%)
Parent ethnicity			
Any white background	98/110 (89.09%)	94/110 (85.45%)	192/220 (87.27%)
African	7/110 (6.36%)	6/110 (5.45%)	13/220 (5.91%)
Asian (Indian, Pakistani, Bangladeshi, any other Asian)	4/110 (3.64%)	6/110 (5.45%)	10/220 (4.55%)
Any other ethnic group	2/110 (1.82%)	4/110 (3.64%)	6/220 (2.73%)
Missing	3/113 (2.65%)	4/114 (3.51%)	7/227 (3.08%)
Parental marital status			
Single	24/111 (21.62%)	22/109 (20.18%)	46/220 (20.91%)
Married and civil partnered	67/111 (60.36%)	64/109 (58.72%)	131/220 (59.55%)
Separated/divorced	4/111 (3.6%)	8/109 (7.34%)	12/220 (5.45%)
Other	16/111 (14.41%)	15/109 (13.76%)	31/220 (14.09%)
Missing	2/113 (1.77%)	5/114 (4.39%)	7/227 (3.08%)
Parent educational qualifications			
None	5/111 (4.5%)	5/110 (4.55%)	10/221 (4.52%)
1-4 passes at CSE, GCSE, O Level	6/111 (5.41%)	4/110 (3.64%)	10/221 (4.52%)
5 or more passes at CSE, GCSE, O level	11/111 (9.91%)	18/110 (16.36%)	29/221 (13.12%)
A Levels or equivalent	36/111 (32.43%)	31/110 (28.18%)	67/221 (30.32%)
Postgraduate degree	50/111 (45.05%)	51/110 (46.36%)	101/221 (45.7%)
Missing	5/113 (4.42%)	5/114 (4.39%)	10/227 (4.41%)

### Attendance at programme sessions

Summary of attendance at programme sessions showed that the mean number of sessions attended in both the LAA and URB arms was 5 out of 8 sessions (62%), ranging from 0 sessions to all eight sessions.

### Fidelity of delivery of LAA and URB programme

Analysis of fidelity of leader’s delivery of the programmes to the manual was undertaken for both arms on 10% of randomly selected session recordings assessed by four independent raters. Sessions were selected for rating from face-to-face delivery (pre-COVID-19 restrictions) and online delivery (LAA: eight face-to-face and seven remote vs URB: nine face-to-face and six remote). For both programmes, the fidelity of delivery was excellent: LAA overall percentage agreement between the independent raters was 93.6% (κ = 0.846), 92.7% for delivery (κ = .897) and 94.5% for content. For URB, overall percentage agreement was 94.9% (κ = .91), 97.2% for delivery (κ = .963), and 90.1% for content.

### SAE outcomes and ESI

Five SAEs were reported across the study: three in URB (two parent related/one child) and two in LAA arm (one parent/one child). None were considered related to taking part in the study or attending the programmes (see Supplementary Material for recorded details of SAE and descriptions of ESI recorded before the 24-week primary study endpoint).

### Primary analysis

Comparing improvement between arms according to CGI-I at 24 weeks

The primary analysis (Table 5) shows the estimate of odds ratio of improvement between the two arms (LAA/URB). Primary analysis was adjusted for the effects of gender, ethnicity and age.

**Table 5.** Summary of analysis on CGI-I.

CGI-I	LAA	URB	URB vs LAA	
	n/N (%)	n/N (%)	Odds ratio (95% CI)	p^a^
Improved	10/81 (12.35%)	11/74 (14.86%)	1.39 (0.52 to 3.69)	0.51
Not improved	71/81 (87.65%)	63/74 (85.14%)		

a p-value obtained from generalised estimating equation with binomial distribution.

**Figure 2.**
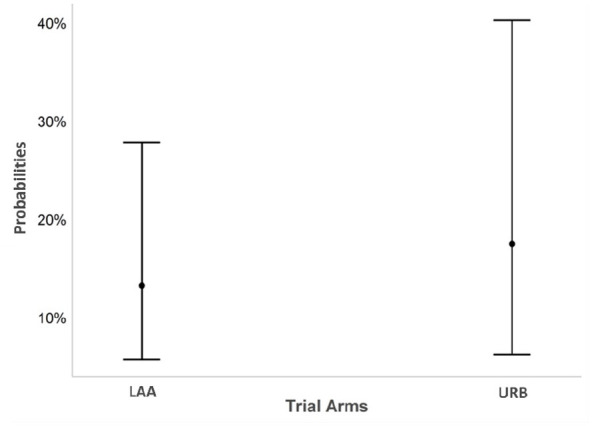
Predicted probabilities of improvement in trial arms.

The result shows that the odds of improvement on average was 39% higher in the URB arm compared to LAA (odds ratio = 1.39; 95% confidence interval (CI) = 0.52 to 3.69; p = 0.51). However, the result is not statistically significant, that is, the confidence interval is sufficiently wide to contain any differences favouring either URB or LAA. Please see Figure 2 for predicted probabilities of improvement in trial arms.

### Pre-specified secondary analysis

#### Analyses of TBV, improvement and impact on families

TBV change scores (in behaviour and impact, responder vs non-responder) were compared between the arms across time points.

Table 6 shows the results of the analysis of change in the TBV at 10, 24 and 52 weeks. Under ‘Positive response in behaviour improvement’, the odds of improvement on average were 92% higher in the URB arm compared to LAA (odds ratio = 1.92; 95% CI = 1.10 to 3.39; p = 0.02) at 24 weeks (primary endpoint). Similarly, for ‘Positive response in impact on family’, the URB arm included significantly more responders than LAA at 24 weeks (odds ratio = 2.10; 95% CI = 1.14 to3.92; p = 0.02).

**Table 6.** Analysis of the TBV (p-value for significant effects are in bold).

	LAA	URB	Total		
Variable	n/N (%)^a^	n/N (%)^a^	n/N (%)^a^	URB vs LAA: odds ratio (95% CI)	p^b^
Positive response in behaviour improvement					
10 weeks	17/151 (11.26%)	40/136 (29.41%)	57/287 (19.86%)	3.26 (1.76 to 6.26)	**0.00**
24 weeks	28/148 (18.92%)	40/132 (30.3%)	68/280 (24.29%)	1.92 (1.1 to 3.39)	**0.02**
52 weeks	31/150 (20.67%)	38/135 (28.15%)	69/285 (24.21%)	1.52 (0.88 to 2.64)	0.14
Positive response in impact on family					
10 weeks	14/151 (9.27%)	27/136 (19.85%)	41/287 (14.29%)	2.4 (1.21 to 4.94)	**0.01**
24 weeks	21/148 (14.19%)	33/132 (25%)	54/280 (19.29%)	2.1 (1.14 to 3.92)	**0.02**
52 weeks	27/150 (18%)	29/135 (21.48%)	56/285 (19.65%)	1.23 (0.68 to 2.22)	0.49

a n is number of responders and N is number of available data.

b p-values are obtained from logistic regression model.

Comparison of the change scores on the TBV between arms at 10 weeks shows that in the URB arm, participants were more likely to have a ‘Positive response in behaviour improvement’ and ‘Positive response in impact on family’ compared with LAA. There was no evidence of any differences between the arms at 52 weeks for all the TBV measures.

### Secondary analysis on continuous outcomes

For secondary analysis of continuous outcomes data, see supplementary material.

#### Differences between and within arms in secondary outcomes

There are few statistically significant differences between the URB and LAA arms in relation to changes in the child secondary outcomes (see Supplementary Table for details). A significant difference is found between the URB and the LAA arm on the Repetitive Behaviour Questionnaire–2 (RBQ-2) Motor Sensory Behaviour subscale score at 10 weeks compared to baseline. However, it is noteworthy that in the LAA arm, the RBQ-2 Motor Sensory Behaviour score on average increased significantly at 10 weeks compared to baseline (+0.09; 95% CI = 0.03 to 0.16), which may account for this difference between the arms, rather than reflecting a reduction in scores for the URB group. For RBQ Teacher Mean Total score, a significant difference is also noted between the arms at 52 weeks compared to baseline. This is most likely accounted for by a decrease in the LAA arm at 52 weeks compared to the baseline. Turning to the parent-reported measures, the parent self-efficacy (PSE) score changed significantly at 10 weeks compared to baseline (+0.34; 95% CI = 0.09 to 0.6) comparing URB arm with LAA, with evidence of more parental self-efficacy in URB compared with the LAA arm. There were no other between-arm differences at any timepoint on any of the secondary outcomes.

Changes over time within both arms can be seen in relation to the VABS-3 subscales at 24 weeks indicating some improvement in parent-reported adaptive functions for autistic children in both arms. However, these changes, although statistically significant, are small and do not represent a clinically meaningful change (Clinch et al., 2023). Changes are also apparent in a number of parent measures (PSE, Autism Parenting Stress Index (APSI) and Warwick–Edinburgh Mental Wellbeing Scale (WEMWBS)) and family functioning measured by the Autism Family Experience Questionnaire (AFEQ). The results indicate that parents in both arms may have benefitted from the programme they received. These changes are largely maintained to 52 weeks.

### Health economic results

The results of the CEA analysis are shown in Table 7. As this table shows, URB is on average more costly and more effective than LAA. There is an incremental cost per additional child achieving the target difference of over £35,000 for URB compared with LAA.

**Table 7.** Costs effectiveness analysis of the children achieved target CGI-I.

Data	Intervention	Cost (£) (CI)	Δ Cost (£)	Children achieved target CGI-I	Δ Children achieved target CGI-I	ICER (Δ cost/Δ children achieved target) (£) CGI-I
Complete case data (n = 136)	LAA (n = 71)	£2655 (£1803–£3507)		0.13		
	URB (n = 65)	£3389 (£2359–£4417)	£734	0.15	0.02	£36,700

Δ: difference; CIs: confidence interval; ICER: incremental cost-effectiveness ratio; LAA: learning about autism; URB: Understanding Repetitive Behaviours; QALY: quality adjusted life year.

### Complete case CUA using QALYs based on responses to the EQ-5D-5L

The results of the complete CUA using QALYs based on responses to the EQ-5D-5L are shown in Table 8. The results show that the URB arm is on average more costly than LAA. The confidence intervals for the adjusted difference in costs have both positive and negative values, meaning that there is also a possibility that URB could be both more or indeed less costly compared with LAA. For QALY, there is, on average, additional QALYs for URB compared with LAA. The confidence interval, however, includes zero. Given the very small average increase in QALYs and more substantial increase in cost per patient on average for URB compared with LAA, the point estimate for the incremental cost per QALY gained for URB compared with LAA was almost £700,000. This estimate does not reflect the imprecision in estimates of costs of QALYs. To illustrate this, as described in section ‘Methods’, bootstrapping of incremental costs and QALYs was conducted, and these were used to plot how incremental costs and QALYs might vary. For the majority of plots (85%), URB is more costly. The estimates of QALYs are evenly distributed (for 53% of the bootstraps URB provides more QALYs). This imprecision is reflected in the cost-effectiveness acceptability curve (presented in tabular form), which shows that URB is unlikely to be considered cost-effective compared with LAA over all the threshold values for society’s willingness to pay for an additional QALY.

**Table 8.** Costs utility analysis of the complete case data. QALYs based on response to the EQ-5D-5 L or CHU9D as indicated in the data column.

Data	Intervention	Unadjusted cost (£) (CIs)	Adjusted Δ cost (£) (CIs)	Unadjusted QALY (EQ-5D-5L) (CIs)	Adjusted Δ QALY (EQ-5D-5L) (CIs)	ICER (Δ cost/Δ QALY) (EQ-5D-5L) (£)	Probability URB cost-effective at different threshold values for society’s willingness to pay for a QALY			
							£0	£20,000	£30,000	£50,000
Cost utility analysis for the EQ-5D-5L Complete case (n = 129)	LAA (n = 68)	£2725 (1838 to 3611)		0.79 (0.74 to 0.83)			85%	78%	73%	67%
	URB (n = 61)	£3394 (2307 to 4480)	£685 (−525 to 1895)	0.83 (0.79 to 0.87)	0.001 (−0.04 to 0.043)	£685,000	15%	22%	27%	33%
Cost utility analysis of the EQ-5D-5L Imputed case (n = 199)	LAA (n = 101)	£2644 (2028 to 3261)		0.78 (0.75 to 0.82)			84%	63%	56%	46%
	URB ( = 98)	£3005 (2287 to 3723)	£445 (−387 to 1278)	0.84 (0.79 to 0.87)	0.01 (−0.02 to 0.04)	£44,500	16%	37%	44%	54%
Data	Intervention	Unadjusted cost (£) (CIs)	Adjusted Δ cost (£) (CIs)	Unadjusted QALY (EQ-5D-5L) (CIs)	Adjusted Δ QALY (EQ-5D-5L) (CIs)	ICER (Δ cost/Δ QALY) (EQ-5D-5L) (£)	Probability URB cost-effective at different threshold values for society’s willingness to pay for a QALY			
							£0	£20,000	£30,000	£50,000
Cost utility analysis of the CHU9D Complete case (n = 120)	LAA (n = 62)	£2394 (1701 to 3087)		0.82 (0.80 to 0.84)			97%	93%	90%	85%
	URB (n = 58)	£3250 (2205 to 4440)	£922 (−1567 to 2001)	0.81 (0.79 to 0.83)	0.001 (−0.002 to 0.023)	£922,000	3%	7%	10%	15%
Cost utility analysis of the CHU9D Imputed case data (n = 181)	LAA (n = 90)	£2450 (1946 to 2954)		0.82 (0.80 to 0.83)	−0.005 (−0.022 to 0.012)	LAA is dominant	88%	90%	89%	87%
	URB (n = 91)	£2822 (2068 to 3576)	£444 (−265 to 1256)	0.80 (0.79 to 0.82)			12%	10%	11%	13%

Δ: difference; CIs: confidence interval; ICER: incremental cost-effectiveness ratio; LAA: learning about autism; URB: Understanding Repetitive Behaviours; QALY: quality adjusted life year.

## Discussion

To our knowledge, this is the first large-scale RCT to investigate the clinical and cost-effectiveness of a parent-mediated programme designed to help parents recognise, understand and respond sensitively to impactful RRB in young autistic children – a research priority highlighted by parents of young autistic children (Autistica Autism, 2017). The primary outcome (the CGI-I) provided a standardised framework of overall improvement rather than simply assessing changes in the specific behaviours targeted using the programme – corresponding to a further priority of the autism community that interventions and measures evaluate real-world changes in the daily lives of autistic individuals (Roche et al., 2021). Both programmes were delivered by practitioners based in the community rather than in specialist centres. The fidelity of delivery of both programmes (initially face to face and then online to comply with the changes necessitated by the COVID-19 pandemic) was high, suggesting that having the possibility of implementing both face-to-face and/or online delivery may increase options in a variety of ways for both parents and healthcare professionals.

Results from this study indicated that there were no differences between arms on the CGI-I, a global clinical judgement of improvement. However, in common with many early intervention evaluation trials, we did see a significant difference between arms on a more proximal outcome (Sandbank et al., 2020). Specifically, children in the URB arm were more likely to be rated by experts as showing improvement in the targeted impactful RRB, and the (negative) impact on the family was also improved relative to the comparison arm. This finding suggests that URB is more effective at supporting parents to understand and reduce the impact of a specific impactful RRB than a psychoeducation programme (LAA). Therefore, although URB was not successful, relative to a generalist psychoeducation group, in producing significant global improvements, it did have a positive effect on the harmful target behaviour selected by parents. In the context of wanting to recognise the utility and joy that autistic people derive from their routines, interests and repetitive movements, this narrower impact of URB only on a specific selected behaviour of interest may help avoid the harm risked by interventions that aim to have a broadly modifying effect on autistic children (Dawson & Fletcher-Watson, 2022).

In terms of assessing generalisation, we did not observe any change in any outcome measures related to potential generalisation. One key aim of the URB intervention was to help parents to recognise RRB, differentiate those that are impactful from those that are not and provide parents with skills to reduce engagement in potentially impactful RRB while not seeking to reduce those that are neutral or potentially helpful/pleasurable. Potential generalisation is therefore more likely to be through the parents in terms of their knowledge, skills and strategies, which in turn may then have a downstream impact on what is observed for the child but is likely to be a slow burn. The lack of impact related to potential generalisation may reflect our choice in timing of when we collected outcome measures, perhaps our choice of a 24-week primary endpoint was too early to detect evidence of broader change in everyday contexts. A future area of research would helpfully include exploration of other possible distal outcome measures. Identifying appropriate measures for evaluating relevant benefits of complex interventions is likely in turn to facilitate the characterisation and evaluation of different aspects of potential generalisation.

The results of the economic evaluation suggest that the URB programme is unlikely to be cost-effective compared with the LAA. These findings were consistent over all the analyses conducted. Furthermore, there was clinically meaningful improvement in parent-reported (self-efficacy, stress and wellbeing) and family functioning across both arms, with no evidence of differences between arms. This indicates that both programmes can be beneficial and supportive for parents, perhaps partially attributable to their shared parent group format. This is in keeping with the emerging evidence base for the efficacy of parent-mediated interventions for autistic children (for a summary of this literature, see reviews by Tan et al., 2024 and Deniz et al., 2022).

In light of a lack of differences between programmes at a global level, benefits of both for parent and family wellbeing, a positive and targeted effect of URB on impactful RRB and the economic differential between the two approaches, clinical services need to make a judgement based on the needs of autistic children and their families in their service. In cases where impactful RRB are prevalent and causing distress, the URB programme has the potential to extend the range of early interventions available to meet the needs of young autistic children and their families, ensuring best the use of therapeutic resources and reducing the potential risk that impactful RRB may cause harm to children.

### Strengths and limitations

A strength of this study is the use of a standardised child outcome measure of overall ‘global’ improvement, the CGI-I. This is in keeping with the research goals of the autism community that measures should more helpfully focus on real-world changes in their daily lives. The lack of evidence of a difference between the two arms at the primary endpoint, perhaps questions the suitability of the primary outcome to detect a difference, as we do find a significant difference between the two arms in relation to the TBVs, the most proximal measure. However, it may also reflect the choice of 24-week primary endpoint as being too early to detect evidence of broadening real-life impact, or that URB’s targeted approach to a single impactful RRB does not result in more expansive changes in the child. However, this was not maintained at 52 weeks, perhaps reflecting changes in circumstances and contexts for the impactful RRB or a wash out of treatment effect. We still have a lot to learn about how changes in RRB might affect global functioning. Future research would benefit from consideration of what to include in a standardised set of outcome measures and what is the optimal primary end-point to detect meaningful change?

The study did not recruit to target, and experienced high attrition at follow-up. Despite employing a number of retention strategies such as consideration of parent burden, flexibility in completion of measures, reminders and study newsletters. Furthermore, delivery of the trial was significantly impacted by the onset of COVID-19. The fidelity of delivery of the programmes in both in-person and online formats was high, but it was not possible to determine whether the change of delivery methods impacted on the high attrition rate reported at follow-up. It is therefore important that future research considers how recruitment and retention of families can be improved. It is difficult to determine the full impact of COVID-19 on the families who were part of the study. We know that families with autistic children faced additional challenges in light of Covid-19 with reduced access to support and services. We heard from parents that many children found changes to their usual structure/routine very challenging, particularly when not able to access familiar strategies (e.g. running around park, visiting favourite places etc). While the move to remote delivery of the study did not seem to have impacted significantly on total recruitment to the trial (although recruitment at the Edinburgh and Lothian’s site was closed), it is apparent, however, that a number of families were lost to follow up. It is therefore difficult to determine the full impact of COVID-19 on the families who were part of the study. Lockdown and changing circumstances for many children may have impacted in un-anticipated ways on many of the outcome measures.

In addition, when considering the approach taken for the economic evaluation there are also limitations. For example, the valuations that underpin the estimation of QALYs for both the EQ-5D and the CHU9D only reflect health on the day that the measurements were completed. Brazier et al. (2019) noted that there can be important elements of the health that are not fully encompassed within the EQ-5D-5L (or CHU9D) including wellbeing and capabilities. Thus, facets of the URB intervention, such as reassurance for the child and caregivers and the ability to plan the future and may not be fully captured by health-related quality of life measure. Instruments that are being developed such as the ICECAP questionnaire for children and young people (ICEpop CAPability CYP) may prove to be a useful addition to the traditional QALY approach in future research (Mitchell et al., 2021). The time horizon for this study is also a consideration, given the follow-up occurs until 52 weeks, which may not be long enough to capture how changes in a child’s behaviour may affect them into adulthood. Future studies could focus on long term outcomes, which would allow valuation of the costs and benefits over a longer period. It should also be noted that the study population came from Scotland and the northeast of England and that the challenges that families of children with autism may differ between communities. Future research could include families from different backgrounds and geographic locations to explore how comparable these groups are when evaluating strategies to help repetitive behaviours.

## Conclusion

This study found no differences between the two parent programmes on a global child outcome measure. However, the results showed that children whose parents attended the URB programme were more likely to have improvements in the targeted, impactful RRB. In addition, parents attending either programme reported clinically meaningful improvement in parent (self-efficacy, stress and wellbeing) and family functioning. However, URB was not likely to be cost-effective compared with LAA within 52 weeks. Future research can focus on outcomes over a longer time horizon to understand the longer-term impact of such interventions and whether these offset the initially higher costs of URB compared with LAA.

The study reconfirms that it is important that clinicians consider both RRB and social communication needs of autistic children with parents when planning appropriate support. We propose that support for parents of young autistic children should maximise the positive impacts of RRB while being sensitive to any potential negative consequences. In the absence of targeted interventions for RRB, the URB programme may be a useful addition to the clinical repertoire. We will continue to work with the autism community, clinical colleagues and researchers to consider the clinical and research implications of this study and promote a wider understanding of RRBs for autistic children and their families.

## Supplemental Material

sj-docx-1-aut-10.1177_13623613251333175 – Supplemental material for Understanding Repetitive Behaviours: A clinical and cost-effectiveness, multi-site randomised controlled trial of a group for parents and carers of young autistic children Supplemental material, sj-docx-1-aut-10.1177_13623613251333175 for Understanding Repetitive Behaviours: A clinical and cost-effectiveness, multi-site randomised controlled trial of a group for parents and carers of young autistic children by Victoria Grahame, Ashleigh Kernohan, Ehsan Kharati, Ayesha Mathias, Chrissie Butcher, Linda Dixon, Sue Fletcher-Watson, Deborah Garland, Magdalena Glod, Jane Goodwin, Saoirse Heron, Emma Honey, Ann Le Couteur, Leila Mackie, Jessica Maxwell, Lewis Montgomery, Emmanuel Ogundimu, Helen Probert, Deborah Riby, Priyanka Rob, Leanne Rogan, Laura Tavernor, Luke Vale, Elspeth Imogen Webb, Christopher Weetman and Jacqui Rodgers in Autism

sj-docx-2-aut-10.1177_13623613251333175 – Supplemental material for Understanding Repetitive Behaviours: A clinical and cost-effectiveness, multi-site randomised controlled trial of a group for parents and carers of young autistic children Supplemental material, sj-docx-2-aut-10.1177_13623613251333175 for Understanding Repetitive Behaviours: A clinical and cost-effectiveness, multi-site randomised controlled trial of a group for parents and carers of young autistic children by Victoria Grahame, Ashleigh Kernohan, Ehsan Kharati, Ayesha Mathias, Chrissie Butcher, Linda Dixon, Sue Fletcher-Watson, Deborah Garland, Magdalena Glod, Jane Goodwin, Saoirse Heron, Emma Honey, Ann Le Couteur, Leila Mackie, Jessica Maxwell, Lewis Montgomery, Emmanuel Ogundimu, Helen Probert, Deborah Riby, Priyanka Rob, Leanne Rogan, Laura Tavernor, Luke Vale, Elspeth Imogen Webb, Christopher Weetman and Jacqui Rodgers in Autism
