# Supplementary material for: Understanding Repetitive Behaviours: A clinical and cost-effectiveness, multi-site randomised controlled trial of a group for parents and carers of young autistic children
Source: Autism. 2025 Jun 9;29(8):1998–2015. doi: 10.1177/13623613251333175 (PMC12255847; doi:10.1177/13623613251333175)
Supplement: sj-docx-1-aut-10.1177_13623613251333175 – Supplemental material for Understanding Repetitive Behaviours: A clinical and cost-effectiveness, multi-site randomised controlled trial of a group for parents and carers of young autistic children [file sj-docx-1-aut-10.1177_13623613251333175.docx]

## Supplementary Materials Health Economics

## Methods

The economic evaluation was carried out using the NICE guidelines for health technology assessment that were applicable when the analysis of the URB data began (National Institute for H, 2013).

## Data Collection

### Intervention Costs

The cost of the URB intervention was estimated from trial data and clinical expert opinion from the trial team. The URB group is delivered by two community-based professionals (facilitators) who have experience working with children with ASD and this was felt to be reflective of how the intervention would be delivered in routine practice. Professional time (the facilitators) required for this intervention was estimated using the clinical expertise of the trial team who designed the intervention. The amount of time and the type of staff required for group facilitations were estimated from expert opinion from the trial team who designed the intervention. Facilitator costs were derived by estimating the time required to prepare and run the group multiplied by the hourly rate for the facilitator based on published estimates (Curtis & Burns, 2020). Printed materials costs were assumed based on trial teams’ estimates of printing rates.

For the LAA arm, the sessions were two hours long and were also delivered by a group facilitator trained and approved by the NAS. The NAS staff rates, the time required to run the group and the printed material costs were all provided by correspondence from the NAS.

For both the URB and LAA arms a per participant cost was derived by dividing the total intervention cost by the average number of participants per group. The number of participants per group was based upon an assumption of nine per group; the most common group size in the Northumberland, Tyne and Wear NHS Foundation Trust which was the site that ran the most groups.

Each randomised participant was allocated a treatment cost based upon which arm they were randomised to.

### Service Utilisation Costs

To measure the services utilised by participants throughout the course of the trial a Service Use Questionnaire (SUQ) was administered to participants for the services that the child used. The SUQ asked participants how many times in the last six months that they have accessed health services for their child. The SUQ was administered at baseline, 24-weeks and 52-weeks follow-up. The SUQ included the number of hospital appointments, inpatient hospital stays (including reason and duration) and the type and number of health care appointments. Appointments differentiated between the different places where appointments were held e.g., participants home; by phone/virtually and within the practice. The SUQ also asked participants to record the medications that they are taking, the duration they had been taking them for and the dose of the medication.

Costs for hospital outpatient appointments, day cases and inpatient stays were obtained from published NHS reference costs (NHS, 2020). Medication costs was estimated from the British National Formulary (BNF) (British National Formulary, 2022).

Costs for community based appointments such as GP or distract nurse appointments were costed using the Unit Costs of Health and Social Care (Curtis & Burns, 2020). Hospital inpatient unit costs were calculated by calculating the number of days that the participant had an inpatient stay by the unit cost derived from NHS reference costs (NHS, 2020). Day case costs were multiplied by the number of days that the participant was in hospital for a day case (Tables 1-4). Medication costs were calculated by multiplying the unit costs of the correct dose of drug by the duration that the participant had been taking it (up to a maximum of six months to match the recall period of each administration of the SUQ.

**Table 1**

*Summary of Inpatient and day case costs*

| **Treatment** | **URB group** | | | **LAA Group** | | |
| --- | --- | --- | --- | --- | --- | --- |
|  | **Mean £ (SD)** | | | **Mean £ (SD)** | | |
|  | 24 weeks | 52 weeks | **Mean** | 24 weeks | 52 weeks | **Mean** |
| Inpatient Stays | 32 (139) | 20(138) | **51 (256)** | 14(95) | 75(590) | **101 (642)** |
| Day Cases | 69 (266) | 99 (379) | **185 (584)** | 21(128) | 81(356) | **70 (331)** |

Table 2

*Unit costs for hospital stays*

| **Item** | **£** | **Unit** | **Reference** | **Notes** |
| --- | --- | --- | --- | --- |
| Inpatient | 378 | Per night | National Reference Costs 2019/2020 v2 (NHS, 2020) | Ward costs per day |
| Day Case Cost | 812 | Per day case | National Reference Costs 2019/2020 v2 (NHS, 2020) | Per day case unit cost. |

Table 3

*Unit costs for appointments*

| Appointments | Unit Cost | Source | Comments |
| --- | --- | --- | --- |
| GP – in Practice | £39.23 | Curtis and Burns, 2020 | Cost per patient contact |
| GP – by Phone | £15.32 | Curtis and Burns, 2020 | GP led triage phone appt |
| GP – at home | £156.00 | Curtis and Burns, 2020 | Based on an hour of General Medical Services |
| GP – Out of hours | £75.74 | Department of Health and NHS England (NHS, 2020) | Based on 2014 values updated to 2020 value. |
| Community Nurse – in practice | £12.67 | Curtis and Burns, 2020 | Based on assumption of 20 min appointment |
| Community Nurse – by phone | £7.62 | Curtis and Burns, 2020 | Nurse led triage phone appt |
| Community Nurse – at home | £43.44 | National Reference Costs 2019/2020 v2 (NHS, 2020) | District Nurse, Adult, Face to face |
| Community Support Worker | £25.00 | Curtis and Burns, 2020 | Per hour of community support worker time |
| Social Worker | £46.00 | Curtis and Burns, 2020 | Per hour, social worker (children) |
| CAMHS | £288 | Curtis and Burns, 2020 | Child And Adolescent Mental Health Services, Outpatient attendance |
| School Nurse | £54.00 | Curtis and Burns, 2020 | School-based children’s health core (other) services – group single professional (one to one) |
| Councillor | £97.00 | Curtis and Burns, 2020 | Per hour, based on an assumption of an hours appointment. |
| Appointments | Unit Cost | Source | Comments |
| NHS 111 Phone line | £9.19 | Turner et al., 2012 | Based on estimate of £8 (2012) adjusted for 2020 costs |
| Psychiatrist | £361.00 | National Reference Costs 2019/2020 v2 (NHS, 2020) | Child and Adolescent Psychiatry, unit costs |
| Psychologist | £201.00 | National Reference Costs 2019/2020 v2 (NHS, 2020) | Clinical Psychology, unit cost |
| Occupational therapist – in practice | £155.00 | National Reference Costs 2019/2020 v2 (NHS, 2020) | Unit Cost, Child one to one. |
| Occupational therapist – at home | £141.00 | Curtis and Burns, 2020 | Community Services - Occupational therapy |
| Paediatrician | £351.00 | National Reference Costs 2019/2020 v2 (NHS, 2020) | Paediatric Neuro-Disability |
| Speech and Language Therapist | £112.00 | National Reference Costs 2019/2020 v2 (NHS, 2020) | Speech and Language Therapist, Child, One to One |
| Accident and Emergency | £113.00 | National Reference Costs 2019/2020 v2 (NHS, 2020) | Accident & Emergency unit cost |

Table 4

*Unit costs for medications*

| **Medication** | **Dose** | **Administration** | **Pack cost (£)** | **Dose Cost (£)** | **Source** | **Comments** |
| --- | --- | --- | --- | --- | --- | --- |
| Atomoxetine | 10mg | Capsule | 21.34 | 0.76 | British National Formulary, 2022 | 28 capsule - tariff price |
|  | 18mg | Capsule | 21.34 | 0.76 | British National Formulary, 2022 | 28 capsules - tariff price |
|  | 40mg | Capsule | 23.52 | 0.84 | British National Formulary, 2022 | 28 capsules - tariff price |
| Mirtazapine | 15mg | Capsule | 1.50 | 0.05 | British National Formulary, 2022 | 28 capsule - tariff price |
| Propranolol | 10mg | Capsule | 1.54 | 0.06 | British National Formulary, 2022 | 28 capsule - tariff price |
| Sertraline | 50mg | Capsule | 1.94 | 0.07 | British National Formulary, 2022 | 28 capsule - tariff price |
| Fluoxetine | 20mg | Capsule | 0.83 | 0.03 | British National Formulary, 2022 | 30 capsule - tariff price |

A per participant cost for resource use was generated for each recall period. Costs at 24 weeks and 52 weeks follow-up were summed so that an overall cost for each participant over the trial follow-up were estimated. Once costs for each participant were derived then a mean cost for each trial arm was generated.

### Patient private care costs and time and Time and Travel Costs

In the CCA, the costs borne by the participants in terms of privately paid for care (Table 5) and time and travel costs for accessing care were also included. Time and travel costs of accessing health care were determining by combining the information for a time and travel questionnaire with information on the use of services.

Table 5

*Unit costs for private therapies*

| Appointments | Unit Cost | Source | Comments |
| --- | --- | --- | --- |
| Homeopathy | £93.94 | Trichard et al., 2004 | Estimate taken from a study managing children with Recurrent Acute Rhinopharyngitis managed by homeopathy. Adjusted to 2020 costs. |
| Traditional Chinese Medicine | £23.00 | University of Westminster, 2022 | Assumption based on provider estimates |
| Private Counselling | £45.00 | Bark, 2022 | Assumption based on provider estimates |
| Private Occupational Therapy | £43.63 | Royal College of Occupational Therapists, 2018 | Cost £42 per session, published 2018 adjusted to 2020 (Campbell and Cochrane Economic Methods Group, 2019) |
| Private Speech and Language Therapy | £70.00 |  | Assumption based on a number of provider estimates |
| Mindfulness | £45.00 |  | Assumed to be the same value as counselling |
| Aromatherapy | £50.00 | Aromatherapy Council, 2022 | Assumption based on provider estimates |

In addition the management of days that the child has taken out of school. Participants were asked if they had to take time away from their work, care or studies to look after a child during school hours. An estimate was used for the number of days that would be taken off school based on a study by Totsika et al. (2020). This study assessed patterns of non-school attendance for those on the autism spectrum. They found a median absence of 2 days per 23 days. This was extrapolated over six months to give a total of 16 days per six-month period follow-up period. A school day was assumed to be six hours, paid time was costed based on the ONS Annual Survey of Hours and Earnings (ASHE) average salary rate (Statistics OfN, 2022). Unpaid time was costed at the leisure time rate based on the study Verbooy *et* al. (2018), hourly estimate value of leisure time and unpaid work.

## Estimation of Effects

### CGI-I Outcomes

The primary effectiveness outcome measure for the cost-effectiveness analysis is achieving at least the target difference in Clinical Global Impression - Improvement scale (CGI-I) at 24 weeks. The outcome is expressed as a percentage of children who achieved their targeted outcome. As noted above, the results for the CEA is expressed as the incremental cost per additional child reaching their target improvement in the CGI-I outcome.

### QALY Outcomes

The primary measure of effects for the CUA is the QALY derived from responses to the EQ-5D-5L in the case of the caregivers and the CHU9D for the children. Both measures were measured at baseline, 24 and 52 weeks for both the EQ-5D-5L and CHU9D. The EQ-5D-5L was completed by the child’s primary caregiver as a measure of their own health related quality of life. The EQ-5D-5L was scored using the EQ-5D-3L value set using the van Hout crosswalk (van Hout et al., 2012). The CHU9D is completed by a proxy on behalf of the child as a measure of their health-related quality of life.

### Analysis of costs and benefits

Continuous and count variables are expressed with appropriate descriptive statistics. This includes means and standard deviations and, for difference in outcome measures, confidence intervals. The relationship between the costs and the outcomes was analysed using a seemingly unrelated regression (SUREG) (Fiebig, 2003). The data was analysed in Stata™ (StataCorp, 2017). Covariates used in the regression include baseline utility measures and care costs in the six months before randomisation.

The price year for costs and benefits was the 2020 in Great British Pounds (GBP), as the cost and benefits for this study were measured within 1 year, no discounting was applied.

### Missing data

For the complete case analysis, only those with complete case data were included. To be included in the complete case dataset all relevant trial instruments had to be completed. This included both the visit and the service use questionnaire had to be noted as complete on the visit record. In addition, to be considered completed the questionnaires must not have had large sections that were not completed (e.g., no values, positive or negative, were given in the entire section). For instruments used to derive (i.e. the EQ-5D-5L or CHU9D) QALY values, data were missing if any one of the three time points of the (baseline, 24 weeks or 52 weeks) was not completed.

To account for those with missing data multiple imputation was carried out. Costs and QALY were imputed using a chained imputation approach using paired mean matching approach using nearest neighbour. This approach was chosen because the data were missing at similar levels and costs and QALYs were considered to be interrelated variables.

## Sensitivity analysis

### Deterministic sensitivity analysis

Deterministic sensitivity analysis was carried out to assess the variability of different parameters on the outcomes of the economic evaluation. One of the key sensitivity analyses was to broaden the cost perspective to be included the costs falling on participants and their families.

### Stochastic sensitivity analysis

To assess the robustness of the study sampling, non-parametric bootstrapping was carried out to assess the uncertainty around the conclusions. Bootstrapping is a technique which resamples a single dataset to create many simulated samples to assess statistical precision. In this case, this meant that the difference in net benefit was estimated for each simulated data set.

In this study, 1000 iterations of the bootstrapping procedure were performed. For the CUA, bootstrapping was carried out for the costs and both the QALY outcomes. The results of the bootstrapping URB were used to develop cost-effectiveness planes. For the CUA the horizontal axis represents the difference in adult QALYs or child QALYs and the vertical axis represents the corresponding difference in costs).

For the CCA appropriate measures of statistical variance e.g., mean and standard deviation were presented.

## Results

### Response rates

The response rates for the SUQ and the TTQ and are summarised in Table 6.

Table 6

*Response rates of Service Use Questionnaire and Time and Travel Questionnaire*

| Questionnaire | LAA Group (n=113) | | | URB Group (n=114) | | |
| --- | --- | --- | --- | --- | --- | --- |
|  | Data Completeness (%) | | | | | |
|  | Baseline | 24 Weeks | 52 Weeks | Baseline | 24 Weeks | 52 Weeks |
| SUQ | 93%  (n=105) | 70%  (n=79) | 72%  (n=81) | 94%  (n=107) | 62%  (n=71) | 65%  (n=74) |
| TTQ | 79%  (n=89) |  |  | 69 %  (n=79) |  |  |
| **Abbreviations:**  LAA = Learning about autism; URB = Understanding Repetitive Behaviours; SUQ – Service Use Questionnaire, TTQ- Time and travel questionnaire | | | | | | |

The response rates for the EQ-5D-5L and the CHU9D are summarised in Table 7.

Table 7

*Data completeness of health-related quality of life questionnaires and Completeness and improvement on the CGI-I scale*

| Outcome | LAA Group (n=113) | | | URB Group (n=114) | | |
| --- | --- | --- | --- | --- | --- | --- |
|  | Data Completeness (%) | | | | | |
|  | Baseline | 24 Weeks | 52 Weeks | Baseline | 24 Weeks | 52 Weeks |
| CHU9D | 81%  (n=92) | 66%  (n=75) | 71%  (n=80) | 82%  (n=93) | 62%  (n=71) | 63%  (n=72) |
| EQ-5D-5L | 92%  (n=104) | 70%  (n=79) | 71%  (n=80) | 89%  (n=102) | 61%  (n=70) | 66%  (n=75) |
| CGI-I Completeness of Data % (n) | 71% (n=81) | | | 65% (n=74) | | |
| Significant improvement in CGI-I score from baseline % (n) | 12% (n=10) | | | 15% (n=11) | | |

**Abbreviations**: LAA = Learning about autism; URB = Understanding Repetitive Behaviours; n = number; SD = standard deviation

### Total resource use

The summary of the use of health services captured by the SUQ are shown below in Table 8.

Table 8

*Appointment and drug costs in each arm*

| Cost Category | LAA Group | | | URB Group | | |
| --- | --- | --- | --- | --- | --- | --- |
|  | Appointments | | | | | |
|  | Mean (£) (SD) | | | Mean (£) (SD) | | |
|  | 24 Weeks | 52 Weeks | Overall Mean | 24 Weeks | 52 Weeks | Overall Mean |
| GP | 25 (37) | 20 (42) | 41 (64) | 36 (56) | 25 (46) | 61 (87) |
| GP Out of Hours | 6 (31) | 6 (34) | 14 (66) | 1 (9) | 2 (12) | 3 (16) |
| Community Nurse | 2 (6) | 2 (10) | 3 (10) | 1 (4) | 11 (76) | 13 (80) |
| Community Support Worker | 1 (8) | 7 (44) | 9 (47) | 3(22) | 2 (12) | 6 (26) |
| Social Worker | 17 (55) | 18 (56) | 30 (101) | 13 (53) | 16 (60) | 31 (95) |
| CAMHS Worker | 55 (191) | 316 (2310) | 406 (2571) | 115 (515) | 163 (529) | 301 (1043) |
| School Nurse | 59 (285) | 139 (1080) | 221 (1333) | 62 (468) | 12 (34) | 17 (47) |
| Councillor | 9 (66) | 2 (15) | 11 (82) | 4 (35) | 7 (46) | 12 (60) |
| NHS 24/ NHS 111 Phone line | 1 (3) | 1 (4) | 2 (4) | 1 (2) | 1 (4) | 2 (5) |
| Psychiatrist | 18 (127) | 18 (97) | 10 (60) | 76 (350) | 112 (405) | 208 (708) |
| Psychologist | 38 (102) | 50 (140) | 82 (178) | 100 (231) | 52 (161) | 161 (350) |
| Occupational therapist | 139 (471) | 121 (383) | 229 (578) | 268 (633) | 269 (852) | 531 (1356) |
| Paediatrician | 187 (291) | 195 (288) | 371 (440) | 243 (412) | 185 (399) | 444 (772) |
| Speech and Language Therapist | 259 (541) | 217 (502) | 448 (764) | 213 (418) | 408 (883) | 629 (1137) |
| Accident and Emergency | 17 (48) | 27 (63) | 35 (85) | 6 (26) | 17 (72) | 26 (93) |
| Other Consultations | | | | | | |
| Cost Category | LAA Group | | | URB Group | | |
|  | Appointments | | | | | |
|  | Mean (£) (SD) | | | Mean (£) (SD) | | |
|  | 24 Weeks | 52 Weeks | Overall Mean | 24 Weeks | 52 Weeks | Overall Mean |
| Other | 83 (278) | 73 (198) | 166 (377) | 72 (227) | 28 (84) | 104 (256) |
| Medications | | | | | | |
| Atomoxetine | 2 (16) | 2 (17) | 2 (17) | 0 (0) | 0 (0) | 0 (0) |
| Mirtazapine | 0 (0) | 0 (0) | 0 (0) | 0 (0) | 0 (0) | 0 (0) |
| Propranolol | 0 (0) | 0 (0) | 0 (0) | 0 (0) | 0 (0) | 0 (0) |
| Sertraline | 0 (0) | 0 (0) | 0 (0) | 0 (0) | 0 (1) | 0 (2) |
| Fluoxetine | 0 (0) | 0 (0) | 0 (0) | 1 (1) | 0 (1) | 0 (2) |
| Other Medications | | | | | | |
| Other | 83 (199) | 94 (202) | 146 (350) | 71 (186) | 88 (262) | 173 (410) |

**Abbreviations**: LAA = Learning about autism; URB = Understanding Repetitive Behaviours; SD = standard deviation

### Health Related Quality of Life

The utility values from both the EQ-5D-5L and the CHU9D Health related quality of life instruments are summarised in Table 10.

Table 10

*Utility Scores in the URB and LAA Groups*

| Outcome | LAA Group (n=113) | | | | URB Group (n=114) | | | |
| --- | --- | --- | --- | --- | --- | --- | --- | --- |
|  | Mean (SD) | | | | Mean (SD) | | | |
|  | Baseline | 24 Weeks | 52 Weeks | QALYs | Baseline | 24 Weeks | 52 Weeks | QALYs |
| CHU-9D | 0.80 (0.11) | 0.81  (0.11) | 0.84  (0.10) |  | 0.78  (0.11) | 0.80  (0.10) | 0.82 (0.11) |  |
| Childs QALYs using CHU-9D (n=121) |  |  |  | 0.82  (0.08) |  | - |  | 0.81 (0.08) |
| EQ-5D-5L | 0.81 (0.19) | 0.82  (0.21) | 0.85 (0.17) |  | 0.73 (0.23) | 0.81 (0.21) | 0.79  (0.21) |  |
| Parents QALYs using EQ-5D-5L (n=130) |  |  |  | 0.79  (0.18) |  |  |  | 0.82  (0.16) |

**Abbreviations**: LAA = Learning about autism; URB = Understanding Repetitive Behaviours; QALY = quality adjusted life year; SD = standard deviation

## Economic evaluation

## Secondary and sensitivity analyses

Table 11 show the imputed sample for the EQ-5D-5L which is a broadly similar picture to the complete case analysis described above. With the larger sample available when data in imputed there is still a slightly larger additional effect on average but at a slightly lower additional average cost for the URB arm compared with the LAA arm. The incremental cost per QALY gained for this analysis is almost £45,000 and the probability that URB would be considered cost effective is no greater than 50% at best over the range of threshold values for society’s willingness to pay for a QALY considered.

*Cost Utility Analysis Using the CHU9D and complete cases*

The results for the complete case CUA using the CHU9D values for the QALYs are summarised in Table 11. The results show a very small effect in favour URB with a larger cost differential compared with the EQ-5D-5L analysis reported above. Overall, the pattern of findings is the same and the URB intervention is unlikely to be considered cost effective over a range of different willingness to pay for a QALY.

Table 11

*Cost Utility Analysis for the secondary analyses and sensitivity analyses*

| Analysis | Data | Intervention | Unadjusted Cost (£) (CIs) | Adjusted ΔCost (£) (CIs) | Unadjusted QALY  (EQ-5D-5L)  (CIs) | Adjusted ΔQALY  (EQ-5D-5L)  (CIs) | ICER (ΔCost/ ΔQALY  (EQ-5D-5L) (£) | Probability URB is cost-effective at different threshold values for society’s willingness to pay for a QALY | | | |
| --- | --- | --- | --- | --- | --- | --- | --- | --- | --- | --- | --- |
|  |  |  |  |  |  |  |  | £0 | £20,000 | £30,000 | £50,000 |
| Cost Utility Analysis of the Imputed Case Data for the EQ-5D-5L | Imputed case data  (n=199) | LAA (n=101) | £2644  (2028 to 3261) |  | 0.78  (0.75 to 0.82) |  |  | 84% | 63% | 56% | 46% |
|  |  | URB ( =98) | £3005  (2287 to 3723) | £445  (-387 to 1278) | 0.84  (0.79 to 0.87) | 0.01  (-0.02 to 0.04) | £44,500 | 16% | 37% | 44% | 54% |
| Cost Utility Analysis Using the CHU9D | Complete case data  (n=120) | LAA (n=62) | £2394 (1701 to 3087) |  | 0.82  (0.80 to 0.84) |  |  | 97% | 93% | 90% | 85% |
|  |  | URB (n=58) | £3250  (2205 to 4440) | £922  (-1567 to 2001) | 0.81  (0.79 to 0.83) | 0.001  (-0.002 to 0.023) | £922,000 | 3% | 7% | 10% | 15% |
| Analysis | Data | Intervention | Unadjusted Cost (£) (CIs) | Adjusted ΔCost (£) (CIs) | Unadjusted QALY  (EQ-5D-5L)  (CIs) | Adjusted ΔQALY  (EQ-5D-5L)  (CIs) | ICER (ΔCost/ ΔQALY  (EQ-5D-5L) (£) | Probability URB is cost-effective at different threshold values for society’s willingness to pay for a QALY | | | |
|  |  |  |  |  |  |  |  | £0 | £20,000 | £30,000 | £50,000 |
| Cost Utility Analysis of the Imputed Case Data for the CHU9D | Imputed case data  (n=181) | LAA (n=90) | £2450  (1946 to 2954) |  | 0.82  (0.80 to 0.83) | -0.005*  (-0.022 to 0.012) | LAA is dominant | 88% | 90% | 89% | 87% |
|  |  | URB (n =91) | £2822  (2068 to 3576) | £444  (-265 to 1256) | 0.80  (0.79 to 0.82) |  |  | 12% | 10% | 11% | 13% |
| Cost Utility Analysis of the Imputed Case Data for the EQ-5D-5L including Time, Travel and Private Costs | Imputed case time, travel & private data (n=199) | LAA (n=101) | £2934  (2290 to 3577) |  | 0.78  (0.75 to 0.82) |  |  | 89% | 76% | 68% | 60% |
|  |  | MRB (n=98) | £3367  (2609 to 4126) | £526  ( -339 to 1392) | 0.83  (0.80 to 0.86) | 0.006  ( -0.027 to 0.039) | £87,667 | 11% | 24% | 32% | 40% |

**Abbreviations**: Δ = difference; CIs = confidence interval; ICER = incremental cost effectiveness ratio; LAA = Learning about autism; URB = Understanding Repetitive Behaviours; QALY = quality adjusted life year

## Cost Consequence Analysis

The results of the CCA are shown in Table 11.

Table 11

*Cost-Consequence Analysis of the LAA and URB intervention*

| Impacts that favour LAA | | Impact that favour URB | |
| --- | --- | --- | --- |
| Measure | Value | Measure | Value |
| RBQ Teacher Mean Total score. URB -LAA change from Baseline at 52 weeks (95% CI). | 0.18 (0.05,0.31) | - | - |
| No evidence of a difference | | | |
| Lower adjusted costs on average (95% CI) | £445 (-387 to 1278) |  |  |
| RBQ2 Sensory Motor Behaviour. URB-LAA. Change from Baseline Diff (95% CI) | -0.01 (-0.09,0.07) | VABS3 Socialization. URB -LAA change from Baseline at 24 weeks (95% CI). | 0.21 (-1.94,2.37) |
| RBQ2 Insistence on Sameness. URB -LAA change from Baseline at 52 weeks (95% CI). | 0.02 (-0.08,0.12) | VABS3 ABC. URB -LAA change from Baseline at 24 weeks (95% CI). | 0.2 (-1.28,1.68) |
| RBQ2 Mean Total score. URB -LAA change from Baseline at 52 weeks (95% CI). | 0.01 (-0.06,0.09) | PSE. URB -LAA change from Baseline at 52 weeks (95% CI). | 0.19 (-0.07,0.44) |
| VABS3 Communication. URB -LAA change from Baseline at 24 weeks (95% CI). | -0.94 (-3.26,1.4) | APSI Total. URB -LAA change from Baseline at 52 weeks (95% CI). | -0.81 (-3.2,1.59) |
| VABS3 Daily Living Skills. URB -LAA change from Baseline at 24 weeks (95% CI). | -0.32 (-2.32,1.65) | AFEQ Experience being parent. URB -LAA change from Baseline at 52 weeks (95% CI). | 0.17 (-1.5,1.85) |
| Impacts that favour LAA | | Impact that favour URB | |
| Measure | Value | Measure | Value |
| WEMWBS. URB -LAA change from Baseline at 52 weeks (95% CI). | -1.3 (-3.45,0.86) | AFEQ Family life. URB -LAA change from Baseline at 52 weeks (95% CI). | -0.04 (-1.39,1.32) |
| AFEQ Total. URB -LAA change from Baseline at 52 weeks (95% CI). | 0.34 (-3.45,4.12) |  |  |
| AFEQ Child development understanding social relationships. URB -LAA change from Baseline at 52 weeks (95% CI). | 0.17 (-1.5,1.85) |  |  |
| AFEQ Child symptoms. URB -LAA change from Baseline at 52 weeks (95% CI). | 0.74 (-0.39,1.86) |  |  |

The time and travel costs are summarised in Table 6 and the school absence costs are summarised in Table 7.

Table 12

*Summary of time, travel and out of pocket costs in each arm*

| **Treatment** | **URB group** | **LAA Group** |
| --- | --- | --- |
|  | **Mean £ (SD)** | **Mean £ (SD)** |
| Travel Costs | 21 (35) | 15 (22) |
| Time Costs | 130 (226) | 95 (198) |
| Private Out of Pocket Costs | 113 (553) | 132 (923) |

Table 13

*Summary of time costs for time spent off school*

| **Treatment** | **URB group (n= 106)** | **LAA Group (n =104)** |
| --- | --- | --- |
|  | **Mean £ (SD)** | **Mean £ (SD)** |
| Time Spent Off School | 497 (573) | 471 (573) |

**References**

Aromatherapy Council. Treatments 2022 [Available from: https://www.aromatherapycouncil.org.uk/treatments

Bark. How much does a Bark. How much does a Therapist cost 2021 2022 [Available from: https://www.bark.com/en/gb/therapy/therapist-prices/Therapist cost 2021 2022 [Available from: <https://www.bark.com/en/gb/therapy/therapist-prices/>

British National Formulary (onlin Bark. How much does a Therapist cost 2021 2022 [Available from: https://www.bark.com/en/gb/therapy/therapist-prices/e) [Internet]. 2022

British National Formulary for C Bark. How much does a Therapist cost 2021 2022 [Available from: https://www.bark.com/en/gb/therapy/therapist-prices/hildren [Internet]. BMJ Group and Pharmaceutical Press. 2022 b [cited 29/03/2022]. Available from: <https://bnfc.nice.org.uk/>.

Campbell and Cochrane Economics Methods Group. EPPI-Centre Cost Converter 2019 [updated 29/04/2019. Available from: https://eppi.ioe.ac.uk/costconversion/

Curtis L, Burns A. Unit Costs of Health and Social Care 2020. Canterbury: University of Kent; 2020

Fiebig, D. G. (2001). Seemingly unrelated regression. A companion to theoretical econometrics, 101-121.

National Institute for H, Care E. Guide to the methods of technology appraisal 2013. London: NICE; 2013

NHS. National Cost Collection: National Schedule of NHS costs - Year 2019-20 - NHS trust and NHS foundation trusts. In: trusts NtaNf, editor. 2020

Royal College of Occupational Therapists. Getting my life back: Occupational therapy promoting mental health and wellbeing in England London: Royal College of Occupational Therapists,; 2018

StataCorp. Stata Statistical Software: Release 15. College Station, TX.: StataCorp LLC.; 2017

Statistics OfN. Annual Survey of Hours and Earnings (ASHE): Business Surveys 2022; 2022 [24/06/2022]. Available from: <https://www.ons.gov.uk/surveys/informationforbusinesses/businesssurveys/annualsurveyofhoursandearningsashe>

Totsika, V., Hastings, R. P., Dutton, Y., Worsley, A., Melvin, G., Gray, K., ... & Heyne, D. (2020). Types and correlates of school non-attendance in students with autism spectrum disorders. Autism, 24(7), 1639-1649.

Trichard, M., Chaufferin, G., Dubreuil, C., Nicoloyannis, N., & Duru, G. (2004). Effectiveness, quality of life, and cost of caring for children in France with recurrent acute rhinopharyngitis managed by homeopathic or non-homeopathic general practitioners: a pragmatic, prospective observational study. Disease Management & Health Outcomes, 12, 419-427.

Turner, J., O’Cathain, A., Knowles, E., Nicholl, J., Tosh, J., Sampson, F., ... & Coster, J. (2012). Evaluation of NHS 111 pilot sites. Final report.

Van Hout, B., Janssen, M. F., Feng, Y. S., Kohlmann, T., Busschbach, J., Golicki, D., ... & Pickard, A. S. (2012). Interim scoring for the EQ-5D-5L: mapping the EQ-5D-5L to EQ-5D-3L value sets. Value in health, 15(5), 708-715.

Verbooy, K., Hoefman, R., Van Exel, J., & Brouwer, W. (2018). Time is money: investigating the value of leisure time and unpaid work. Value in Health, 21(12), 1428-1436.
