# Supplementary material for: Understanding Repetitive Behaviours: A clinical and cost-effectiveness, multi-site randomised controlled trial of a group for parents and carers of young autistic children
Source: Autism. 2025 Jun 9;29(8):1998–2015. doi: 10.1177/13623613251333175 (PMC12255847; doi:10.1177/13623613251333175)
Supplement: sj-docx-2-aut-10.1177_13623613251333175 – Supplemental material for Understanding Repetitive Behaviours: A clinical and cost-effectiveness, multi-site randomised controlled trial of a group for parents and carers of young autistic children [file sj-docx-2-aut-10.1177_13623613251333175.docx]

**Serious Adverse Events Supplementary Table**

|  | **LAA** | **URB** | **Total** |
| --- | --- | --- | --- |
| **Variable** | n/N(%) | n/N(%) | n/N(%) |
| Who For |  |  |  |
| Parent/carer | 0/2(0%) | 2/3(66.67%) | 2/5(40%) |
| Child | 2/2(100%) | 1/3(33.33%) | 3/5(60%) |
| Missing | 0/2(0%) | 0/3(0%) | 0/5(0%) |
| Severity |  |  |  |
| Mild | 2/2(100%) | 0/3(0%) | 2/5(40%) |
| Moderate | 0/2(0%) | 2/3(66.67%) | 2/5(40%) |
| Severe | 0/2(0%) | 1/3(33.33%) | 1/5(20%) |
| Death | 0/2(0%) | 0/3(0%) | 0/5(0%) |
| Missing | 0/2(0%) | 0/3(0%) | 0/5(0%) |
| Causality | | | |
| Yes | 0/2(0%) | 0/3(0%) | 0/5(0%) |
| No | 2/2(100%) | 3/3(100%) | 5/5(100%) |
| Missing | 0/2(0%) | 0/3(0%) | 0/5(0%) |
| hospitalisation |  |  |  |
| Yes | 2/2(100%) | 3/3(100%) | 5/5(100%) |
| No | 0/2(0%) | 0/3(0%) | 0/5(0%) |
| Missing | 0/2(0%) | 0/3(0%) | 0/5(0%) |
| Outcome |  |  |  |
| Recovered | 2/2(100%) | 1/2(50%) | 3/4(75%) |
| Condition improved | 0/2(0%) | 1/2(50%) | 1/4(25%) |
| Missing | 0/2(0%) | 1/3(33.33%) | 1/5(20%) |

**Events of Special Interest Supplementary Table**

| ESI Category | **LAA**  **N** | | **URB**  **N** | |
| --- | --- | --- | --- | --- |
| Study Phase | During Intervention | During Follow-Up Phase | During Intervention | During Follow-Up Phase |
| Moved out of area |  |  | 1 |  |
| School Difficulties |  | 1 | 1 | 2 |
| Financial Difficulties | 1 |  |  |  |
| Parental Stress | 2 | 2 | 2 |  |
| Physical Health (Child) |  |  | 1 |  |
| Physical Health (Parent) |  | 1 | 1 |  |
| Mental Health (Sibling) |  |  | 1 |  |
| Caring Responsibilities (Grandparent) |  | 1 | 1 |  |
| Family Bereavement |  |  | 1 | 2 |
| COVID-19 Physical Health (Family) |  |  |  | 1 |
| COVID -19 Financial Difficulties |  | 1 |  |  |
| COVID -19 Family Separation |  |  |  | 1 |
| **Total per study phase** | **3** | **6** | **9** | **6** |
| **Overall total** | **9** | | **15** | |
